# Pain in the acephate: A case of protracted toxicity after an intentional ingestion of ant killer

**DOI:** 10.1016/j.toxrep.2026.102311

**Published:** 2026-07-09

**Authors:** Max Trojano, Matthew Lippi, Solana Archuleta, Leanne Cook, Daniel Lasoff, Alicia Minns

**Affiliations:** University of California San Diego Health, Department of Emergency Medicine, Division of Medical Toxicology, 200 West Arbor Drive, San Diego, CA 92013, United States

**Keywords:** Acephate, Organophosphate, Insecticide, Atropine, Pralidoxime

## Abstract

Organophosphates (OP) have a variety of applications, including widespread use as pesticides. Although OP poisoning in the U.S. has been declining, toxicity from intentional ingestions of commercially available preparations is still a concern. We describe a case of a 17-year-old female who intentionally ingested an acephate-based fire ant insecticide and developed severe cholinergic toxicity, despite acephate’s classification as a safer OP. The patient was successfully treated with atropine and pralidoxime, but had recurrence of symptoms after long asymptomatic periods requiring repeat dosing, which is atypical. We encourage clinicians to have a low threshold for prolonged observation in these patients, even in the absence of symptoms.

## Highlights

• Severe toxicity from acephate is rarely reported.
• Our patient developed cholinergic toxicity treated with atropine and pralidoxime.
• The patient had atypical symptom recurrence after long asymptomatic periods.
• Extended observation after intentional ingestions of acephate may be warranted.

## Graphical Abstract

**Figure:**
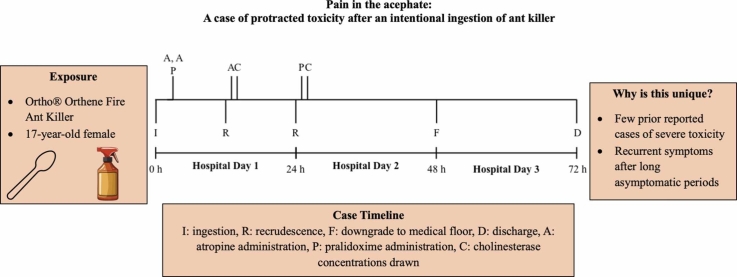

## 1 Introduction

Organophosphates (OP) are a class of structurally similar compounds that are used as pesticides, herbicides, and chemical warfare agents. OPs cause human toxicity by inhibiting acetylcholinesterase (AChE) resulting in an increased concentration of acetylcholine at synapses in both the peripheral and central nervous systems. This produces a cholinergic toxidrome through overstimulation of muscarinic and nicotinic receptors. Organophosphate poisoning in the U.S. has been declining since 1997, which is likely due to the U.S. Environmental Protection Agency’s (EPA) initiative to phase out OPs in residential settings [1]. However, some OPs are still available to and widely used by the public, including acephate (O,S–dimethyl acetylphosphorodithioate), which can cause toxicity in humans.

The World Health Organization classifies insecticides into five groups based on rat LD_50_ for oral exposures. The groups range from Class Ia, which is “extremely hazardous,” to Class U, which is “unlikely to present acute hazard.” Acephate is considered safer than other insecticides as a Class II OP (“moderately hazardous;” LD_50_ 1.0–1.4 g/kg in rats) [2]. Due to its comparative safety profile to other OPs, combined with few reports of significant toxicity in humans, the EPA has suggested relaxing acephate’s environmental constraints [3]. In contrast, we describe a case of an adolescent female who ingested Ortho® Orthene, a fire ant insecticide consisting of at least 50% acephate and additional non-toxic proprietary ingredients, who developed significant toxicity requiring repeated doses of atropine and pralidoxime.

## 2 Case presentation

**Fig. 1:**
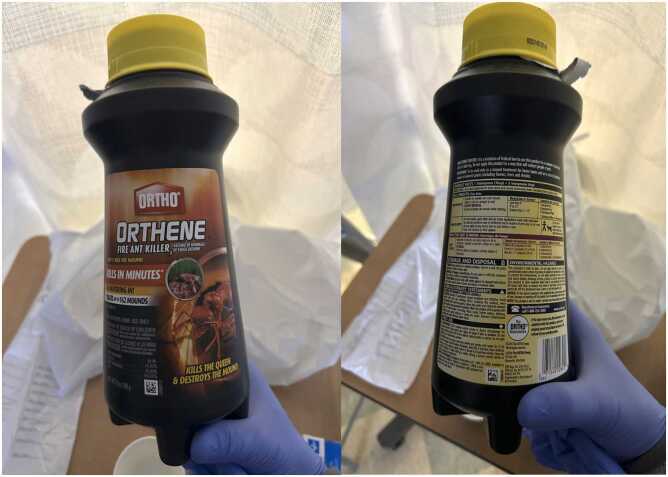
Ortho Orthene® Fire Ant Killer bottle.

This case involved a 17-year-old Pashto-speaking female who initially presented to a community emergency department (ED) after intentionally ingesting Ortho® Orthene Fire Ant Killer. The ingestion occurred approximately two hours prior to the patient’s arrival at the ED. The patient herself admitted the ingestion to her mother shortly after it occurred. The patient’s father was called home from work and found the patient lethargic, diaphoretic, and with copious oral secretions. Emergency medical services (EMS) were called. At the time of the patient’s initial presentation, it was reported that she ingested approximately one half of the bottle (∼ 170 g). The bottle accompanied the patient to the ED via EMS and was indeed noted to be half empty (Fig. 1); however, later in her course the patient clarified that she only ingested one “spoonful."

In the ED, the patient had a temperature of 35.6 °C, blood pressure 116/67 mmHg, heart rate 129 beats per minute, and a respiratory rate 19 breaths per minute with an oxygen saturation of 98% on room air. On examination, the patient was noted to be lethargic and minimally responsive to questioning. She also had copious oral secretions, generalized weakness, and muscle fasciculations. She had no bronchorrhea or bronchospasm. Laboratory studies were largely unremarkable, aside from a slight leukocytosis (13.1 K/mcl), hyperglycemia (146 mg/dL), hypokalemia (3.0 mmol/L), hypomagnesemia (1.8 mg/dL), mildly decreased serum bicarbonate (19 mmol/L), and elevated INR (1.4). Her acetaminophen and salicylate concentrations were undetectable. A comprehensive urine drug screen was also negative. A computed tomography scan of the head, electrocardiogram, and chest x-ray were also obtained and were unremarkable. Poison Control was contacted and recommended giving atropine and pralidoxime. Atropine was titrated to secretion resolution, specifically oral secretions and bronchorrhea, as a clinical endpoint. Importantly, heart rate was not used as a primary definition of atropinization. Although bradycardia is classically associated with the cholinergic toxidrome, tachycardia may predominate in OP poisoning due to stimulation of nicotinic receptors at sympathetic ganglia. As such, using heart rate as a marker of clinical improvement risks underdosing of atropine. The patient received 1 mg of atropine and then a second 1 mg dose approximately 15 min after the first. A bolus dose of 1.5 g of pralidoxime was also administered. These interventions significantly improved both the patient’s oral secretions, generalized weakness, and fasciculations. She did not require any additional treatment while at the community ED. She was then transferred to a specialized pediatric hospital with a dedicated toxicology consultation service.

Approximately 12 h after her ingestion, the patient was evaluated in the pediatric intensive care unit by our toxicology service, where she was found to be tachycardic (heart rate 115), lethargic, and with copious oral and respiratory secretions. She did not have any appreciable weakness or muscle fasciculations, but complained of nausea and generalized abdominal pain. An aliquot of 1 mg of atropine was given with almost immediate improvement in her symptoms. She was started on dextrose and potassium containing maintenance fluids. Serum and red blood cell (RBC) cholinesterase concentrations were obtained, which resulted after hospital discharge.

**Fig. 2:**
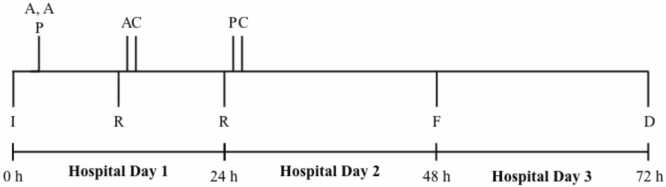
Timeline of clinical course and interventions. The top half of timeline displays interventions, while the bottom half displays clinical events. I: ingestion, R: recrudescence, F: downgrade to medical floor, D: discharge, A: atropine administration, P: pralidoxime administration, C: cholinesterase concentrations drawn.

On reevaluation nearly 24 h after her ingestion, she had no recurrence of her cholinergic symptoms and did not require any additional atropine. She was noted to be more alert and reported feeling near baseline. However, she did have prominent tongue fasciculations. She did not have any proximal muscle or neck flexor weakness. Negative inspiratory force was normal throughout her inpatient stay. Interim labs were remarkable for ongoing hypomagnesemia (1.6 mg/dL) and a mild respiratory alkalosis (7.42/32.5/22.0/80.2). Her repeat INR and CK were within normal limits. Given her tongue fasciculations, the decision was made to administer an additional 1 g of pralidoxime as a bolus. She had no progression of her fasciculations, and by hospital day 2 the patient’s neurological exam had returned to normal. At this time, she was downgraded from intensive care and was monitored on the medical floor for an additional 24 h with no recurrence of symptoms (Fig. 2).

The patient’s initial cholinesterase concentrations, which were obtained 14 h after her ingestion, were notable for a plasma cholinesterase level of 0.2 U/mL (normal 2.9–7.1 U/mL) and RBC cholinesterase of 3.2 U/mL (normal 7.9–17.1 U/mL). A subsequent sample obtained 26 h after ingestion showed improvement of both the plasma cholinesterase (1.5 U/mL) and RBC cholinesterase (8.8 U/mL).

## 3 Discussion

This case highlights the toxic effects and natural history of a non-fatal, intentional ingestion of acephate. Despite initial control of the patient’s symptoms and an asymptomatic period of at least 12 h, the patient developed recurrence of both muscarinic and nicotinic toxicity requiring repeat dosing of atropine and pralidoxime. This clinical course is not typical, as the majority of acephate exposures seem to result from exploratory ingestions in children with only minor clinical effects [4].

Acephate’s unique metabolism in humans likely limits its toxicity. Acephate itself is a weak inhibitor of AChE [5]. Humans metabolize acephate to the more potent OP methamidophos (O,S–dimethyl phosphorodithioate), which is a class Ia OP (“highly hazardous;" rat LD50 15.0–30.0 mg/kg) [2]. This metabolite is thought to cause the majority of toxicity associated with acute acephate exposure. However, humans are believed to have two protective mechanisms that limit acephate’s cholinergic toxicity. First, the carboxyamidase enzyme responsible for the conversion of acephate to methamidophos is inhibited by methamidophos itself creating a negative feedback loop [6]. Second, acephate, through binding at an allosteric site on acetylcholinesterase, may reduce binding of methamidophos at the active site [5].

Despite these protective mechanisms, severe toxicity and death have been reported. These cases all involved intentional ingestions. Jiang et al., describe a case in which a 27-year-old female required intubation, 9 mg of atropine, 2 g of pralidoxime, and subsequent infusions of these medications for four days before resolution of symptoms [7]. However, the acephate preparation and the quantity ingested were not reported, making comparison to our case difficult. Huttner et al. report a Orthene ingestion in a 33-year-old male who required 4 mg atropine and 2 g pralidoxime at the time of his presentation; he had no recurrence of toxicity [8]. Takayasu et al. describe post-mortem concentrations of both acephate and methamidophos in various tissues after an overdose on a 50% acephate solution [9]. Interestingly, the ratio of methamidophos to acephate was highest in the central nervous system, which may help to explain acephate’s ability to cause significant toxicity in large overdoses. This finding has also been replicated in rats injected intraperitoneally with either acephate or methamidophos; the acephate treated rats had significantly higher methamidophos concentrations in brain tissue [10]. The underlying mechanism for acephate’s predilection for the central nervous system is not known.

It is unclear why our patient developed recurrent toxicity, specifically recurrence of isolated nicotinic symptoms after 24 h. It is important to note that methamidophos is a dimethyl OP, which can form an irreversible (i.e., aged) complex with AChE. This aging process has a half-life of approximately 3 h [11]. Oximes can reactivate OP-inhibited AChE, but cannot reverse aged enzymes. Thus, early administration of an oxime is critical to prevent aging, as was done in this case. However, pralidoxime has a relatively short half-life (∼75 min) and its efficacy may have waned over time, allowing acephate and methamidophos to re-inhibit AChE. It is also possible that there may have been ongoing absorption of acephate and subsequent conversion to methamidophos resulting in further AChE inhibition and recurrent toxicity. Both acephate and methamidophos are hydrophilic and it is less likely that there was re-distribution from adipose tissue [3]. In any event, it should be noted that there is no universally accepted dosing regimen for pralidoxime and the repeat dose given in our case was meant to target newly inhibited AChE.

This case has several limitations that constrain inferences regarding exposure level and resulting toxicity. First, the amount of ingested acephate is ultimately unknown; however, it is unlikely that the patient ingested half the bottle as initially reported by EMS. At that time, the patient herself was unable to provide additional details and did not have any family with her to validate the ingested quantity. A language barrier may have also affected the medics’ understanding of the ingested amount on scene. After she had been stabilized, the patient was questioned with the assistance of a translator and refuted this initial report noting that she ingested only one “spoonful.” The patient’s father also remarked that he had previously used about half of the bottle several months prior and did not notice a significant amount missing when presented with the bottle in the hospital. Second, blood and urine concentrations of acephate and methamidophos were not obtained and as a result no toxicokinetic characterizations can be asserted in this case. Cholinesterase activity measurements, while a widely accepted and clinically useful surrogate marker for OP toxicity, are non-specific and do not distinguish between specific OP compounds.

Despite these limitations, this example underscores that severe toxicity can occur despite acephate’s touted safety profile. Atropine and pralidoxime appear to be effective. Our patient also had recurrent toxicity twice during her hospital course after asymptomatic periods. As such, we urge clinicians to have a low threshold for prolonged observation in asymptomatic patients after intentional ingestions.

## CRediT authorship contribution statement

**Max Trojano:** Writing – review & editing, Writing – original draft. **Alicia Minns:** Writing – review & editing, Writing – original draft, Supervision. **Daniel Lasoff:** Writing – review & editing. **Leanne Cook:** Writing – review & editing. **Solana Archuleta:** Writing – review & editing. **Matthew Lippi:** Writing – review & editing.

## Declaration of Competing Interest

The authors declare that they have no known competing financial interests or personal relationships that could have appeared to influence the work reported in this paper.

## Data Availability

No data was used for the research described in the article.
